# An unusual case of capecitabine hyperpigmentation: Is hyperpigmentation a part of hand-foot syndrome or a separate entity?

**DOI:** 10.4103/0253-7613.70401

**Published:** 2010-10

**Authors:** Biju Vasudevan

**Affiliations:** Department of Dermatology, MH Shillong, Shillong, Meghalaya - 793 001, India

**Keywords:** Capecitabine, hand-foot syndrome, hyperpigmentation

## Abstract

A 59-year-old man with adenocarcinoma of stomach was prescribed capecitabine as adjuvant chemotherapy. After two cycles of therapy, patient developed hyperpigmentation on hands and feet. Examination revealed a peculiar distribution of hyperpigmentation on hands and feet and in addition, hyperpigmented spots on the dorsum of tongue. Although hand-foot syndrome (HFS) to capecitabine solely manifesting as palmoplantar hyperpigmentation has been described earlier, this is probably the first instance wherein oral pigmentation has also been found in association. In addition, this finding lends support to the growing argument of hyperpigmentation being a separate entity: different from HFS, both therefore being separate adverse effects of the same drug.

## Introduction

Capecitabine is an oral antineoplastic agent, which is converted to 5-fluorouracil (5-FU) in the body. It is approved by FDA for treatment of colonic, metastatic colorectal, and metastatic breast cancers. The most common dose-limiting adverse effects of capecitabine are hyperbilirubinemia, diarrhea, and hand-foot syndrome (HFS). HFS was first described by Lokich and Moore in 1984 in association with 5-FU.[1] In this study, we present a case of varied palmoplantar hyperpigmentation associated with oral hyperpigmentation as an adverse effect to capecitabine. Such an association has rarely been described in the literature, and it raises a doubt about the current nomenclature of HFS and its etiopathogenes.

## Case Report

A 59-year-old man, diagnosed as adenocarcinoma of the stomach in March 2008 underwent distal radical gastrectomy in January 2009. After radiotherapy for three cycles, the patient was prescribed tablet capecitabine as adjuvant therapy. Capecitabine was prescribed 2 g twice daily for 2 weeks, followed by a week without drug. After two complete cycles of therapy, the patient developed discoloration of the hands, feet, and tongue. He, however, did not complain of redness, swelling, ulceration, pain, or dysesthesia over the discoloured areas. There was no history of similar lesions or reaction to other drugs. Patient did not have similar complaints occurring in any family members.

**Figure 1:**
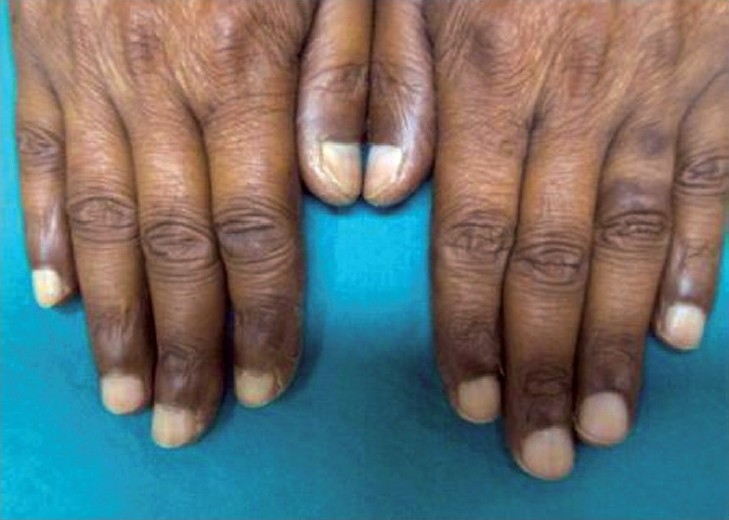
Hyperpigmentation on dorsum of hands.

**Figure 2:**
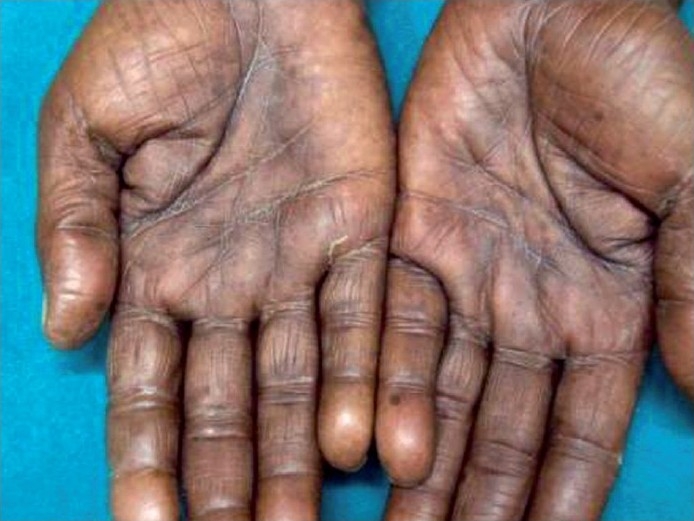
Hyperpigmentation on palmar creases.

**Figure 3:**
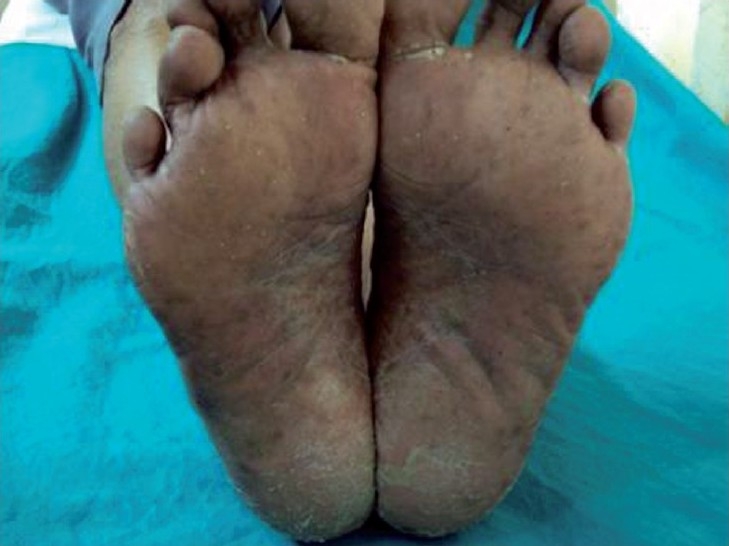
Hyperpigmentation on instep of feet.

**Figure 4:**
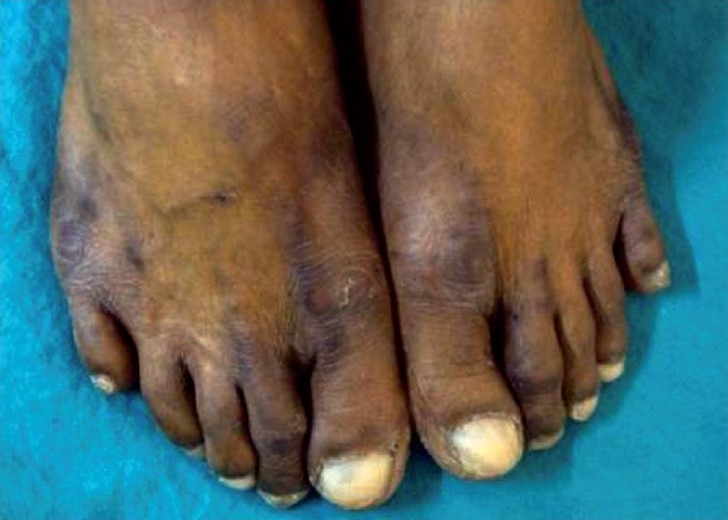
Hyperpigmentation on dorsum of both feet.

**Figure 5:**
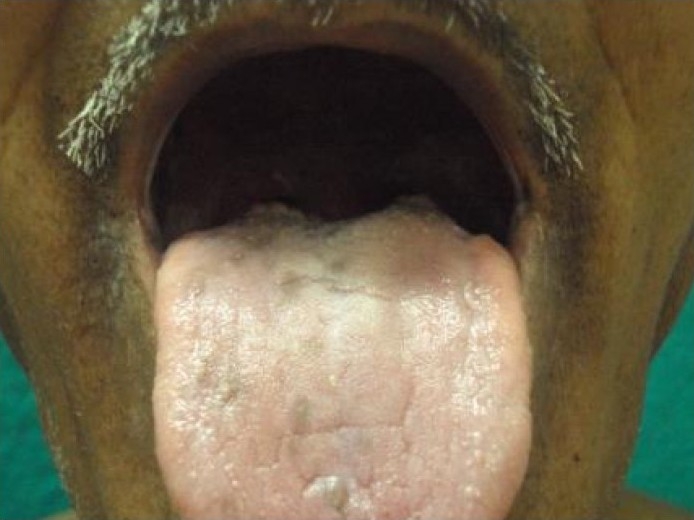
Hyperpigmented spots on tongue.

Examination revealed hyperpigmentation of both hands and feet. The distribution of altered pigmentation was, however, peculiar with involvement of the dorsum of both hands, especially the regions over the interphalangeal and metacarpophalangeal joints [Figure 1]. The creases on the palms were also prominently involved [Figure 2]. Instep of both feet was affected [Figure 3], while the dorsum of feet showed a peculiar distribution of hyperpigmentation [Figure 4]. Cuticles of all nails were ragged, but there were no other nail changes. In addition to the above, hyperpigmented spots were also found on the dorsum of tongue [Figure 5]. The patient was prescribed topical emollients and the condition improved. The drug was, however, continued. Following completion of therapy, all pigmented areas including those on tongue faded.

## Discussion

Capecitabine is a carbamate derivative of 5’-deoxy-5-fluorouracil.[2] It is metabolized to 5-FU via three enzymatic steps with final conversion to 5-FU by thymidine phosphorylase. The most frequent adverse cutaneous reaction associated with capecitabine is HFS. Other adverse effects include fatigue, weakness, abdominal pain, nausea, vomiting, diarrhea, and bone marrow suppression.[3] Drugs which have been associated with HFS include 5-FU, capecitabine, cytarabine, doxorubicin, epirubicin, fluorodeoxyuridine, hydroxyurea, mercaptopurine, cyclophosphamide, docetaxel, and vinorelbine.[4]

HFS, also known as palmoplantar erythrodysesthesia, is classified into three grades: Grade 1 consists of erythema with swelling, dysesthesia or paresthesia, Grade 2 is a progression where pain and discomfort affect the daily activities of the patient, and Grade 3 is the superimposition of blistering, moist desquamation and ulceration, coupled with severe pain. However, hyperpigmentation of hands and feet, rather than erythema are found to be the initial manifestation in most patients and are considered by many authors as Grade 1 HFS.[5,6]

Various theories have been put forward for the etiopathogenesis of HFS: increased expression of thymidine phosphorylase, which converts capecitabine to active 5-FU in keratinocytes leading to inflammation,[7] excess collection and secretion of the drug by eccrine sweat glands which are highly concentrated on the palms and soles,[8] and the latest research findings which claim that small-fiber neuropathy is the likely cause of neuropathic symptoms encountered in the condition. Mounting evidence suggests that HFS may be caused by products of dihydropyrimidine dehydrogenase (DPD)-initiated catabolic degradation of 5-FU.

Variations of HFS syndrome mentioned in the literature include keratoderma-like thickening, scleroderma-like changes, longitudinal melanonychia, and paronychia.[9] A case of atypical HFS with hyperpigmentation of palms alone has been described in an African patient which also suggests that pattern of cutaneous manifestations may vary in patients with different ethnic backgrounds.[10] There has been a report earlier of corneal pigmentation in the eye due to the drug. However, it is for the first time in the literature that a case of oral pigmentation as a side effect of capecitabine is being described along with HFS. Also, the hyperpigmentation on hands and feet was distributed in a unique manner compared to all previous reports.

It is an interesting finding that neither the grading system nor the etiopathogenesis explains the hyperpigmentation associated with the syndrome. Whether hyperpigmentation is a part of the HFS is yet to be determined. If the neural theory holds good, then hyperpigmention alone as a side effect of the drug will have to be treated as an entity, separate from HFS. This is substantiated by our case wherein oral pigmentation was present, which questions the nomenclature and etiopathogenesis of the condition.

In this case, HFS occurred in the patient after two cycles of capecitabine taken for adenocarcinoma. The pigmentation was not present prior to therapy and no other drugs were taken prior to capecitabine. In addition, the patient also had oral pigmentation. Following completion of treatment, the pigmentation regressed. This confirms that the HFS with oral hyperpigmentation could be attributed to capecitabine.
